# Retraction Note: Enhancement of novel *Endo-polygalacturonase* expression in *Rhodotorula mucilaginosa PY18* : insights from mutagenesis and molecular docking

**DOI:** 10.1186/s12934-025-02885-9

**Published:** 2025-12-18

**Authors:** Nagwa M. Abd El-Aziz, Maysa E. Moharam, Nora N. El-Gamal, Bigad E. Khalil

**Affiliations:** 1 Microbial Genetic Department, Biotechnology Research Institute, National Research Centre, 33 El Buhouth St, Dokki, Cairo, 12622 Egypt; 2 Microbial Chemistry Department, Biotechnology Research Institute, National Research Centre, 33 El Buhouth St, Dokki, Cairo, 12622 Egypt

**Retraction: Microbial Cell Factories (2023) 22:252** 10.1186/s12934-023-02253

The Editor-in-Chief has retracted this article because it was mistakenly accepted without peer review. Additionally, Fig. 12 PY18 and E-54 lanes appear highly similar, and the authors have not provided the underlying raw data for validation.

Nagwa M. Abd El-Aziz does not agree with this retraction. Maysa E. Moharam, Nora N. El-Gamal and Bigad E. Khalil have not responded to any correspondence from the editor or publisher about this retraction.

